# The Indirect Effect of Future Anxiety on the Relationship between Self-Efficacy and Depression in a Convenience Sample of Adults: Revisiting Social Cognitive Theory

**DOI:** 10.3390/jcm13164897

**Published:** 2024-08-19

**Authors:** Marta Szota, Aleksandra M. Rogowska, Aleksandra Kwaśnicka, Karolina Chilicka-Hebel

**Affiliations:** 1 Institute of Psychology, University of Opole, 45-040 Opole, Polandaleksandra.kwasnicka@uni.opole.pl (A.K.); 2 Department of Health Sciences, Institute of Health Sciences, University of Opole, 45-040 Opole, Poland

**Keywords:** depression, DFS-5, emerging adulthood, future anxiety, GSES, middle-aged adults, PHQ-9, self-efficacy, social cognitive theory

## Abstract

**Background/Objectives**: Depression and anxiety are prevalent disorders, particularly during emerging adulthood. Uncertainty about the future, exacerbated by unstable times, can lead to heightened future anxiety in this group. This study aimed to examine the complex associations of depression symptoms, future anxiety, and self-efficacy in adults from Poland. Additionally, we investigated age and gender differences in depression symptoms, future anxiety, and self-efficacy. **Methods:** A cross-sectional online survey study was performed in 2023 in Poland using snowball sampling. A convenience sample of 284 adults participated in this study, ranging in age between 18 and 65 years old (*M* = 32.18, *SD* = 11.87), including 95 men (33.45%) and 189 (66.55%) women, and also 157 (55.63%) emerging adults (18–28 years old) and 126 (44.37%) middle-aged adults (29–65 years old). The Patient Health Questionnaire (PHQ-9), Dark Future Scale (DFS-5), and Generalized Self-Efficacy Scale (GSES) were used to measure depression, future anxiety, and self-efficacy, respectively. **Results:** A 2-way ANOVA showed that both emerging adults and women scored significantly higher in depression symptoms and future anxiety than middle-aged adults and men, respectively. Furthermore, women scored lower in self-efficacy than men. Analyses revealed that there was a positive correlation between depression and future anxiety. Self-efficacy was negatively correlated with depression and future anxiety. Self-efficacy and future anxiety accounted for 48% of depression variance, controlling for age and gender. Future anxiety was found to be a partial mediator of the relationship between self-efficacy and depression. **Conclusions:** This study significantly advances the understanding of mental health in adults, grounded in social cognitive theories, revealing that low self-efficacy heightens future anxiety, thereby exacerbating depression symptoms in the Polish adult population, independent of age and gender. Emerging adults and women need psychological support to reduce depression and future anxiety. Women, in particular, should be the main focus of interventions to boost self-efficacy. Implementing targeted preventive measures and support systems can mitigate the challenges faced by emerging adults and women.

## 1. Introduction

### 1.1. Depression in Emerging Adults

Depression is one the most common mental disorders, which is characterized by such symptoms as low mood, feelings of emptiness, hopelessness, sadness, loss of interest, apathy, sleep, and appetite disturbances. According to the World Health Organization [1], 3.8% of the population suffers from severe depression symptoms globally, including 5% of adults. A higher risk of depression was found in women than in men [1,2,3,4,5,6]. Studies showed that the risk of depression increases during emerging adulthood [2,7,8]. Numerous studies found higher depression symptoms in early adulthood compared to other developmental periods [9,10,11,12,13,14,15,16]. Emerging adulthood is a transitional phase between adolescence and adulthood, during which individuals commonly engage in an exploration of various roles and possibilities. Emerging adulthood, originally defined by Jeffrey Arnett in 2000, is the developmental period between adolescence and full adulthood [17,18]. While it was initially characterized as spanning from 18 to 25 years old [17,18], it is now recognized as extending up to 29 years [7]. The emerging adulthood stage results from delays in assuming traditional adult roles, such as starting a family, and prolonged education leading to a later career start [19]. Research [17] identified five distinctive features of this period: identity explorations, instability, self-focus, feeling in-between, and possibilities or optimism. These attributes highlight the intense and challenging process of identity formation, which can provoke complex and challenging thoughts about the future, stress, and uncertainty, which may heighten the likelihood of depression. Indeed, research showed that the first incidence of major depressive disorder is the lowest in childhood compared to subsequent periods but the highest during the emerging adulthood period, with 51% of cumulative incidence [4]. A longitudinal study examined trajectories of depressive symptoms in young adults during the ten years and found a low level of symptoms in 23% of the participants and a moderate level among 61% of the sample, and 16% presented high and increasing levels during emerging adulthood [20]. Furthermore, the risk of depression increased, especially during the COVID-19 pandemic, among emerging adults [21,22,23,24]. Therefore, it is essential to fully explain the phenomenon of high levels of depressive symptoms among emerging adults [2,8,25,26,27].

### 1.2. Association between Future Anxiety and Depression

Depression frequently co-occurs with somatic symptoms and anxiety disorders [12,13,28,29,30]. A meta-analysis showed that depression is bidirectionally related to anxiety [31]. However, anxiety symptoms more strongly predicted depressive symptoms than vice versa [31,32,33]. Anxiety may concern worry or fear about the future [34,35]. 

A negative view of the future can be seen as a symptom of depression or the core causal element of depression [36,37]. Future anxiety (FA) was defined by Zaleski [38] as a state of apprehension encompassing uncertainty, fear, worry, and concern for unfavorable changes in one’s more remote personal future. It can be considered a specific attitude about the future in which negative cognitive and emotional processes are predominant [38]. Despite the name suggesting a great emotional load, FA has a strong cognitive component, and the author even suggests it is a personality characteristic based on the subjective future time perspective [38]. Thoughts and imagination about the future can provoke FA, especially in emerging adults, whose futures are still in the making [39]. A lower level of FA has been found among men than among women [40,41], which is consistent with findings about anxiety in general [29]. Research confirmed that depressive symptoms are more prevalent among those with high FA [33,42]. 

### 1.3. The Relationship between Depression, Future Anxiety, and Self-Efficacy

Self-efficacy is one of the personal resources mitigating the detrimental effects of various hardships such as anxiety, stress, and an uncertain future. Self-efficacy, as defined in social cognitive theory, is a set of beliefs about one’s capability to influence one’s life [43,44,45,46]. Believing in the ability to influence the circumstances motivates actions and choices and determines effort and perseverance [44,47], resulting in lower anxiety toward the outcome, whether it is near or far [39]. There are inconsistencies in research regarding age and gender differences in self-efficacy and competence beliefs. Still, research suggests that younger people and women score lower on these variables than older adults and men, even though they have similar achievements [48,49,50]. 

Research shows a negative correlation between future anxiety and self-efficacy [51]. Furthermore, studies consistently show a medium [52,53] or strong negative relationship between self-efficacy beliefs and depression [54,55,56]. This relationship appears to be stronger in females than in males [52]. Research showed that low self-efficacy is a significant predictor of high depression symptoms in undergraduates from the USA [3]. However, the direction of influence is not apparent here and is a topic of current discussion [53,57,58]. Furthermore, systematic research showed that there is a gap in research on the relationship between self-efficacy and depression among emerging adults [59].

The theory of learned helplessness explains the development of depression as a result of low self-efficacy levels and an actual or perceived lack of control over the outcome of a situation [60,61]. People with learned helplessness tend to attribute the source of the problem to themselves, generalize the problem to many aspects of life, and perceive it as persistent or permanent. Low self-efficacy causes a strong belief in the inability to effectively perform a specific task or cope with difficulties, which leads to depressed mood and amotivation [36,60,61,62].

### 1.4. The Current Study

Although there is little research on the selective association between future anxiety, self-efficacy, and depression, there is a lack of comprehensive research that explains the complex associations between the three variables in a single sample, especially among young adults. This research will fill the gap by comparing a group of emerging adults with middle-aged adults in terms of self-efficacy, future anxiety, and depression. The main goal of this research will be to find the mechanism explaining depression in young and middle-aged adults. Beck’s cognitive theory [63,64] is one of the most important psychological theories explaining depression development and integrating all three variables: depression, future anxiety, and self-efficacy. The cognitive theory assumes that in the early stages of life, beliefs are formed that affect our perception of the world and ourselves. People with depression have non-adaptive and dysfunctional information processing and beliefs. Depression results from the negative cognitive triad, in which the occurrence of critical or traumatic life events activates prejudiced thoughts, which leads to automatic negative thoughts about oneself, as well as about past, present, and future events. Cognitive errors maintain dysfunctional assumptions, and negative self-schemas limit the reception of information to those that correspond to this schema. Indeed, research indicated a positive association between future anxiety and depression [65,66,67,68]. Studies found evidence that the poor evaluation and generation of possible futures and negative beliefs about the future can drive depression [36,65,69,70]. 

MacLeod et al. [32,71] examined retrospective and prospective thinking in participants with anxiety and depression disorders. The study found that patterns for recall of past experiences and anticipation of future experiences were very similar. Therefore, a sense of self-efficacy, which is based mainly on past experiences and social feedback, can affect future thinking, leading to increased depression. Another research [72,73] showed that both episodic and semantic components of autobiographical memories and imagined future events may lead to maladaptive changes in perceived self-efficacy in combat veterans who have post-traumatic stress disorder (PTSD). In contrast, enhancing self-efficacy in combat veterans improves episodic thinking about the future and promotes cognitive strategies associated with positive mental health outcomes [74]. However, research examining complex relationships between self-efficacy, future anxiety, and depression, especially in emerging adults, is still missing.

Based on previous studies and integrating Beck’s cognitive theory of depression [63,64,75] with Bandura’s social cognitive theory [43,44,45,46,76], we assume the following hypotheses:

1. There are age (emerging and middle adulthood) and gender (women, men) differences in symptoms of depression, future anxiety, and self-efficacy (H1).
2. Self-efficacy is related negatively to symptoms of depression and future anxiety (H2).
3. The higher the level of future anxiety, the higher the level of depression symptoms (H3).
4. Low self-efficacy contributes to higher depression directly and indirectly through increased levels of future anxiety (H4).

## 2. Materials and Methods

### 2.1. Study Design and Procedure

The cross-sectional research was conducted using online Google Forms. The link to the survey was shared on private social media profiles and on Facebook groups, which allowed the snowball method to be used. The inclusion criteria were to be adults between 18 and 65 years old. This study was anonymous and voluntary. The Assessment Committee approved the research project at the Institute of Psychology of the University of Opole (decision No. KOJBN 30/2023, 5 December 2023). The survey began with an informed consent form, and only those who consented were allowed to participate. The data were collected between 3 July 2023 and 6 December 2023.

The sample size was determined a priori using G*Power ver. 3.1.9.7. For the 2-way analysis of variance (ANOVA), a minimum *N* = 269 people were expected, considering *p* < 0.05 (α), power 0.80 (1 − β), and medium effect size (ƒ = 0.25). The required sample size was *N* = 65 for linear multiple regression (fixed model, R^2^ increase) if assuming five tested predictors, *p* < 0.05 (α), power 0.80 (1 − β), and medium effect size (ƒ^2^ = 0.15). The minimum sample size for structural equation modeling was *N* = 145, calculated as 5 cases for each item (a total of 24 items were included in the model). Initially, 291 people responded to the invitation, but two of them did not consent to participate, and five were excluded due to being over 65 years old. Hence, the final sample comprised 284 people. Post hoc power analysis showed that a sample of *N* = 284 participants (including 157 emerging adults and 126 middle-aged adults) indicates a power of 0.83 for ANOVA and 0.99 for regression analysis in this study.

### 2.2. Participant Characteristics

Participants were 284 adults from Poland, including 95 men (33.45%), aged between 18 and 64 years old (*M* = 32.18, *SD* = 11.87). Participants were divided into two age groups: 157 (55.63%) emerging adults (18–28 years old) and 126 (44.37%) middle-aged adults (29–65 years old). Most participants (28.17%) live in a medium-sized city (between 50,000 and 250,000 inhabitants), have higher education (26.76% have a Bachelor’s degree, while 28.17% have a Master’s degree), are in a relationship (69.01%), are employed (52.47%), and have good economic status (45.07%). Distributions of various sociodemographic variables in the two samples are presented in Table 1.

### 2.3. Measures

#### 2.3.1. Depression

The 9-item Patient Health Questionnaire (PHQ-9) was used to screen for symptoms of depression [77,78] based on the Diagnostic and Statistical Manual of Mental Disorders, 4th edition (DSM-IV). The scale consists of nine questions related to individual symptoms of depression. Participants use a 4-point response scale to assess the severity of depression symptoms (0 = Not bothering me at all, 1 = A few days, 2 = More than half the days, and 3 = Almost every day). The higher the total score (ranging from 0 to 27), the greater the severity of depression. A score of 0 to 4 means no depression, 5 to 9 mild depression, 10 to 14 moderate depression, and 20 to 27 severe depression. In this study, the reliability of the PHQ-9 was assessed by Cronbach’s α = 0.87.

#### 2.3.2. Future Anxiety

The Dark Future Scale (DFS) was used to assess the symptoms of future anxiety [41]. This is a shortened, 5-item version of the 29-item Future Anxiety Scale [79]. Participants respond on a 7-point Likert scale (0 = Definitely false, 1 = False, 2 = Rather false, 3 = Difficult to say, 4 = Rather true, 5 = True, and 6 = Definitely true). The higher the sum of the scores (ranging from 0 to 30), the higher the fear of the future. The internal consistency in the current study was Cronbach’s α = 0.90.

#### 2.3.3. Self-Efficacy

The 10-item Generalized Self-Efficacy Scale (GSES) was used to measure self-efficacy [80,81]. The strength of one’s belief in their ability to cope with difficult circumstances was rated on a 4-point Likert scale (1 = Not true at all, 4 = Exactly true). A sum of scores (ranging from 10 to 40) indicates the magnitude of self-efficacy. Internal consistency in the current study was Cronbach’s α = 0.91.

#### 2.3.4. Demographic Survey

The demographic information included questions about gender (Women, Men, Other), age (number of years old), the place of residence (village, small town up to 50,000 inhabitants, medium-sized city from 50,000 to 250,000 inhabitants, large city from 250,000 inhabitants up to 500 thousand inhabitants, and a very large city over 500 thousand inhabitants), education (Primary, Vocational, Secondary, Bachelor degree, Master degree), relationship status (Single, In a relationship), the professional status (Student, Student employed, Employed, Unemployed), and economic status (Insufficient, Enough for basic needs, Meets more than basic needs).

### 2.4. Statistical Analysis

A preliminary analysis of descriptive statistics were conducted, including a number of items (Items), range of scores (Range), mean (M), standard deviation (SD), skewness (Skew.), kurtosis (Kurt.), Cronbach’s alpha (α), McDonald’s omega (ω), and Pearson’s r correlations. A 2 (Age: Emerging and Middle-aged adults) x 2 (Gender: Men and Women) ANOVA was performed to examine the effect of age and gender on depression, future anxiety, and self-efficacy. The Bonferroni post hoc test was implemented to assess significant differences between particular groups and to estimate the effect size of Cohen’s *d* and partial eta-square ($\eta_{p}^{2}$). Next, the hierarchical multiple linear regression analysis was performed for depression symptoms as an outcome variable. Age and gender were included in the regression model in the first step, self-efficacy in the second step, and future anxiety in the third step. Finally, a covariance-based structural equation modeling (CB-SEM), with the Robust Weighted Least Squares (WLSMV) estimation method, was performed in this study to verify the hypothesis about the mediation model [82]. Composite-based statistics, including average variance extracted (AVE), square root of the AVE, and heterotrait-monotrait (HTMT), were used to examine the convergent and discriminant validity of the structural model [82,83]. Construct validity was evaluated also using several goodness-of-fit criteria [84], including ML χ^2^, *df*, and *p*-value (the ratio χ^2^/*df* < 2 is considered very good fit, between 2 and 3—good, and acceptable < 5), standardized root mean squared residual (SRMR ≤ 0.08 is acceptable), root mean square error of approximation (RMSEA; acceptable fit if ≤0.08, adequate fit if <0.06, and good if 0.04), and comparative fit index (CFI is acceptable if ≥0.90, and good if >0.95). The measurement invariance (MI) was examined using multigroup SEM (MGSEM) to check the moderating effect of age and gender on the mediation model in the latent variable and particular items. Ref. [85] suggests a change of ≤–0.005 in CFI, supplemented by a change of ≥0.010 in RMSEA, as an indicator of non-invariance when the compared sample sizes are unequal. For testing intercept or residual invariance, a change of ≥–0.005 in CFI, supplemented by a change of ≥0.010 in RMSEA, would indicate non-invariance. All statistical tests were performed using the JAMOVI software ver. 2.3.28.

## 3. Results

### 3.1. A Preliminary Statistical Analysis

Descriptive statistics were examined to assess the validity and reliability scores of the constructs measured in this study (Table 2). Since the sample size was medium (*N* < 300), and skewness and kurtosis ranged between ± 1, we assumed that the criteria of a normal distribution were met [86]. Therefore, parametric tests were performed in the later steps of statistical analyses. Pearson’s correlation showed that self-efficacy was related moderately and negatively to both future anxiety and depression symptoms, which confirmed the hypothesis 2. Moreover, moderate and positive correlations were found between future anxiety and depression symptoms, confirming the hypothesis 3. Reliability (McDonald’s ω) was high for all variables (self-efficacy, future anxiety, and depression symptoms), ranging from 0.88 to 0.90.

As shown in Table 2, a convergent validity showed that AVE was higher than the recommended value of 0.50 [82] in self-efficacy, future anxiety, and depression symptoms, indicating that these three variables share a high degree of variance and are meaningfully interrelated. Furthermore, the square root AVE for each construct was higher than the construct’s correlations with other scales, ensuring appropriate discriminant validity between these three variables [83]. In addition, the heterotrait-monotrait (HTMT) ratio of correlations ranged between 0.62 and 0.76, suggesting an acceptable level of discriminant validity (HTMT < 0.85), which reflects the extent to which a construct better explains the variance in its own indicators, compared to the variance of other constructs [82]. Therefore, the convergent and discriminant validation was confirmed in the mediation model.

### 3.2. Gender and Age Differences in Depression Symptoms, Future Anxiety, and Self-Efficacy

A 2-way ANOVA was conducted to examine group differences in depression symptoms, future anxiety, and self-efficacy. Table 1 presents mean scores and standard deviation for each variable by age and gender. The hypothesis 1 (H1) for depression was confirmed, as a significant effect of age was found on depression, with emerging adults scoring higher in depression symptoms than middle-aged adults (*p* < 0.001, Cohen’s *d* = 0.44), but the effect was small for the main effect, *F*(1, 280) = 11.75, *p* < 0.001, $\eta_{p}^{2}$ = 0.04. Also, gender was a significant factor in depression, with higher depression symptoms among women than men (*p* < 0.001, Cohen’s *d* = –0.58), with medium effect size for main effect, *F*(1, 280) = 20.54, *p* < 0.001, $\eta_{p}^{2}$ = 0.07. However, interaction effect was insignificant, *F*(1, 280) = 0.12, *p* = 0.73, $\eta_{p}^{2}$ = 0.00. 

Similarly, the H1 for future anxiety was also confirmed. A main effect of age was presented on future anxiety, with emerging adults scoring higher in future anxiety than middle-aged adults (*p* < 0.01, Cohen’s *d* = –0.34). However, the effect size for the main effect of age on future anxiety was marginal, *F*(1, 280) = 6.93, *p* < 0.01, $\eta_{p}^{2}$ = 0.02. Significantly higher levels of future anxiety were shown in women than men (*p* < 0.001, Cohen’s *d* = –0.53), with medium effect size for the main effect of gender on future anxiety, *F*(1, 280) = 17.20, *p* < 0.001, $\eta_{p}^{2}$ = 0.06. Interaction between age and gender was insignificant, *F*(1, 280) = 0.17, *p* = 0.68, $\eta_{p}^{2}$ = 0.00.

Finally, the H1 for self-efficacy was only partially confirmed. ANOVA showed that emerging adults do not differ from middle-aged adults in self-efficacy (*p* = 0.20, Cohen’s *d* = 0.20), *F*(1, 280) = 2.45, *p* = 0.119, $\eta_{p}^{2}$ = 0.01. However, men scored significantly higher in self-efficacy than women (*p* < 0.001, Cohen’s *d* = 0.86), with a large effect size for the main effect of gender on self-efficacy, *F*(1, 280) = 45.16, *p* < 0.001, $\eta_{p}^{2}$ = 0.14. The interaction effect between age and gender on self-efficacy was not found in this study, *F*(1, 280) = 0.73, *p* = 0.39, $\eta_{p}^{2}$ = 0.00.

### 3.3. Associations between Depression, Future Anxiety, and Self-Efficacy

The hierarchical regression analysis showed that both young age and gender are significant predictors of depression symptoms, explaining 12% of depression variance (Table 3). When self-efficacy was included in the regression model in the second step, gender was no longer a significant predictor of depression. Still, low self-efficacy levels and emerging adulthood were significant predictors of depression. Explained variance increased to 33%, indicating that self-efficacy contributes 21% to depression variance. In the third step, future anxiety was included in the regression model, showing three significant predictors of depression: young age, low sense of self-efficacy, and high future anxiety levels. All variables explain 48% of depression variance, including 15% of future anxiety selective variance. The magnitude of self-efficacy decreased in the third step, indicating that future anxiety contributes to the relationship between self-efficacy and depression, playing a potential mediating role in this association.

### 3.4. Analysis of Mediation

**Figure 1:**
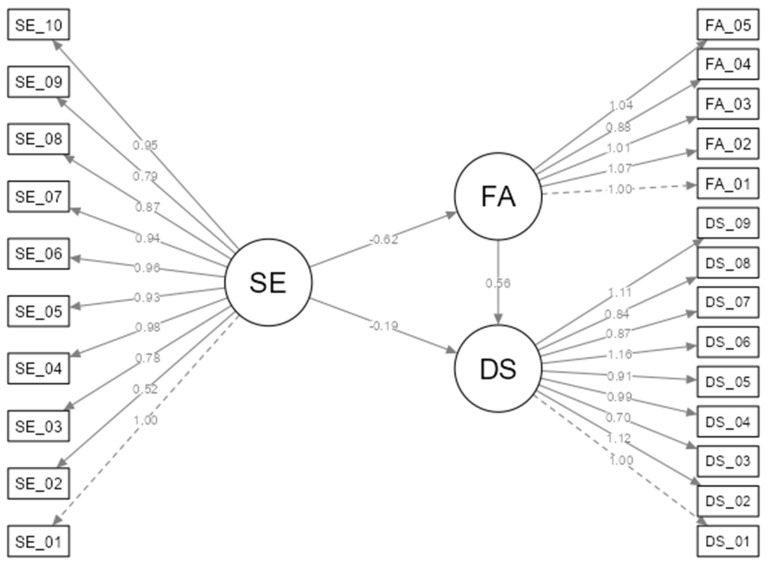
A mediation model showing the indirect effect of self-efficacy (SE) on depression symptoms (DS) through future anxiety (FA).

The mediating effect of future anxiety on the relationship between self-efficacy and depression was examined using structural equation modeling (SEM) analysis (Table 4 and Figure 1). The mediation model showed acceptable fit indices for χ^2^/*df* = 1.25, *p* = 0.005, RMSEA = 0.029 (95% CI = 0.017, 0.040), SRMR = 0.053, and CFI = 0.99 [84]. All associations were significant, confirming that future anxiety partially mediates the relationship between self-efficacy and depression symptoms in adults, which confirms the hypothesis H4 (Table 4 and Figure 1). 

The measurement invariance is a hierarchical test, assuming more equality restrictions in each consecutive model in the following sequence: configural, metric, scalar, and strict. Configural invariance verified whether the same mediation structure is valid in each age or gender group. 

The multigroup SEM (MGSEM) showed configural invariance for age since fit indices improved compared to the baseline model: χ^2^/*df* = 1.02, *p* = 0.38, RMSEA = 0.011 (95% CI = 0.000, 0.031), SRMR = 0.069, and CFI = 1.00. Similarly, configural invariance was considered for gender because fit indices were better than in the baseline model, χ^2^/*df* = 0.59, *p* = 1.00, RMSEA = 0.000 (95% CI = 0.000, 0.000), SRMR = 0.061, and CFI = 1.00. A good multi-group model fit suggests that the overall mediation model holds up similarly for all ages and genders. Therefore, neither age nor gender moderates the mediation model.

## 4. Discussion

This study examined for the first time the complex associations between depression, future anxiety, and self-efficacy based on social cognitive theories. Most of our hypotheses were confirmed, which is discussed in detail in the following sections.

### 4.1. Age and Gender Differences in Depression, Future Anxiety, and Self-Efficacy

Consistent with hypothesis 1 (H1), we found some age and gender differences in depression, future anxiety, and self-efficacy. In particular, emerging adults showed greater symptoms of depression and future anxiety than middle-aged adults, although the effect size was small for these age differences. Our study confirms a large body of the previous literature that emerging adulthood is a risk factor for developing depression [2,3,4,5,8,10,11,20,21,22,23,25,26,27,87]. During the early adulthood period, many stressful life problems accumulate. Young people do not cope well, experimenting and learning new ways of solving cognitive, social, professional, personal, and interpersonal problems [7,17,18,88]. The unstable and unpredictable global situation, combined with the challenges commonly faced by young adults, contributes to increased anxiety about their future. The difference in mental health between emerging adulthood and middle-aged adults may be affected by the levels of instability in family and professional spheres. Middle-aged adults often exhibit a higher sense of self-efficacy, attributed to the goals they have achieved and the experiences they have gained, which fosters a belief in their ability to manage difficulties. In contrast, depressive disorders among young people may stem from the postponement of traditional adult roles and the belief that they are unable to cope with developmental tasks, leading to increased future anxiety. Indeed, previous studies showed that future anxiety increases during early adulthood [39]. 

The present research showed that women scored higher in depression symptoms and future anxiety and lower in self-efficacy than men, and the effect size was from medium to large for these gender differences. Consistent with previous studies, a higher risk of depression is systematically and globally demonstrated in women compared to men [2,3,4,5]. Studies also showed higher levels of anxiety symptoms, including future anxiety, among women than men [29,40,41]. Many factors contribute to these gender differences [6]. Women more often present with internalizing symptoms (increasing anxiety and depressive mood), whereas men present with externalizing symptoms. Also, changes in ovarian hormones during lifespan may contribute to the increased prevalence of depression in women. Women also frequently suffer from premenstrual dysphoric disorder, postpartum depression, and postmenopausal depression and anxiety. In addition to biological factors, women are more susceptible to depression than men due to social risk factors, including differences in physical strength and personality traits, as well as social attitudes that promote inequality and mental health disparities between the sexes [6]. 

A current study showed that men experience higher self-efficacy and lower future anxiety and depression, which is consistent with previous research [3]. Although some studies found inconsistencies in research regarding age and gender differences in self-efficacy, younger people and women usually scored lower on self-efficacy than older adults and men [48,49,50]. Compared to men, women may rely more on social information than on their prior achievements to build self-efficacy and face unique self-esteem barriers, believing in lower levels of self-efficacy and competence than men [48]. However, more research is required to fully understand gender and age differences in self-efficacy, as well as in future anxiety and depression.

### 4.2. The Relationships between Depression, Future Anxiety, and Self-Efficacy

We assumed that self-efficacy is related negatively to symptoms of depression and future anxiety (H2), and future anxiety is related positively to depression symptoms (H3). These hypotheses were fully confirmed in this study. The correlation analysis confirmed both hypotheses, which is consistent with the current literature. For example, previous research found a negative relationship between self-efficacy and future anxiety [51] and depression [3,52,53,54,55,56], showing that self-efficacy is a powerful asset lowering the risk of mental problems following the problematic circumstances. Self-efficacy may influence affective states through the inability to control emerging negative thoughts, which also appear in depression [89]. When someone believes they are unable to manage their own life and has a low sense of self-efficacy, depression is likely to ensue. Possessing a considerable degree of self-efficacy empowers individuals to adeptly navigate anxiety by feeling adequately prepared for potential difficulties and dispelling fear-laden thoughts.

A positive association between future anxiety and depression found in this study is also in line with previous research [42,65,67,68], and confirm learned helplessness theory [36,60,61,69]. A review study indicated that future-oriented thinking plays a crucial role in the development of depression disorders [65]. Research showed that an increased tendency to anticipate adverse events in the future is related positively to anxiety and depression symptoms severity [65,66,67,68]. Future anxiety can be considered either a symptom of depression or the core causal element of depression [36,37,38]. Research confirmed a positive correlation between future anxiety and depression measured by Beck’s scale [42]. Future anxiety is defined as an attitude about the future in which negative cognitive and emotional processes predominate. Individuals feel anxious about undesirable situations [41]. Negative thoughts and imaginations about the future are enough to cause future anxiety, and failed attempts to get rid of these thoughts could increase anxiety and depression [33,38,39,42,90,91,92]. However, the relationship between depression symptoms and faulty prospection can be bidirectional, as depression symptoms may perpetuate faulty prospection and contribute to a self-defeating cycle. Therefore, Roepke and Seligman [36] propose the use of future-oriented treatment strategies based on cognitive behavioral therapy to improve prospection and mitigate depression symptoms.

Both future anxiety and self-efficacy beliefs are derived from the memory of past experiences and the cognitive appraisal of current situations. Future anxiety is negatively correlated with self-efficacy [51]. Zaleski [38] suggested that the cognitive nature of future anxiety is strongly associated with self-efficacy, according to the social learning theory [44,45]. Anxiety occurs before situations that may harm a person. Self-efficacy gives a person the confidence to cope with difficulties, which reduces the sense of threat. Suppose persons believe that they cannot manage challenges; anxiety increases and can lead to depression [43,46]. Threats related to global disasters and conflicts may cause fear of the future [38,42,90,91,92]. Gambin et al. [87] showed that high scores for future anxiety and depression increased during the COVID-19 pandemic. The COVID-19 pandemic has caused many changes in people’s daily lives, and most people have been unable to leave their homes. Isolation has a negative impact on a person’s emotional state, especially since social contact is an important part of life when entering adulthood. Any activities performed outside the home carried the risk of contracting the virus. Additionally, information about the statistics of infected people and mortality rates appeared in the media, which could have caused an increase in fear. The mental effects of the pandemic did not end when the epidemiological state was lifted. Also, wars taking place around the world, especially the Russian invasion of Ukraine, beyond Poland’s eastern border, and the worsened economic situation related to global inflation may contribute to the deterioration of mental health among Poles.

### 4.3. Mediating Effect of Future Anxiety on the Relationships between Self-Efficacy and Depression

A hierarchical regression model was built to explain depression among emerging adults, controlling for gender and age, with future anxiety and self-efficacy as predictors. The results highlighted the role of self-efficacy in safeguarding one’s mental health. Aside from self-efficacy, age and future anxiety were found to be significant predictors, explaining together 48% of the variance in depression. Based on previous findings and social cognitive theory, we assumed that low self-efficacy contributes to higher depression directly and indirectly through increased levels of future anxiety (H4). The SEM analysis confirmed the partial mediating effect of future anxiety on the relationship between self-efficacy and depression. Furthermore, the mediation model was universal and independent of age and gender. 

The present study can be fully explained within a social cognitive theory [43,44,45,46,63,64,75,76,93]. According to the social learning theory, Bandura [45,76] identified four basic properties of human agency: intentionality, forethought, self-reactivity, and self-reflection. Forethought involves future-oriented plans based on established goals. Cognitive visualization of the future plays a crucial role in promoting purposeful and predictable behavior as a timely guide and motivator. A well-thought-out perspective gives direction, coherence, and meaning to life [76]. The social cognitive theory of self-regulation [43,44,46] emphasizes the role of self-efficacy in initiating behaviors, persevering in their implementation, emotions, and feelings experienced during this process, coping with obstacles and failures, feeling stress, re-evaluating goals, modifying methods of action and satisfaction after completing the task. The belief in self-efficacy refers to one’s competencies, properties, preferred social and moral values, and the acceptability and inadmissibility of behavior. In this perspective, motivation to engage in a given behavior is related to prediction processes, the decision to undertake the activity with self-efficacy, and the assessment of effects with evaluation processes in which emotions, feelings, beliefs, and values play a significant role [43,44,45,46]. Motivation is based on anticipation. Anticipation is a complex system of variables, including assessing the value of the goal and the chances of success, estimating the necessary effort, costs, and sacrifices, predicting the feelings and thoughts that will appear during the actions, as well as the well-being and self-esteem after the actions are performed [44,47]. Anticipation also has a strong affective component, both positive (hope, excitement) and negative (concerns, worries, and anxiety). Therefore, the social cognitive theory thoroughly explains the association between future anxiety and self-efficacy.

Consistent with Beck’s cognitive theory of depression [63,64,75], Hoerger et al. [67] found associations between depressive symptoms and dysphoric forecasting bias, which can be understood as the tendency of individuals in dysphoric states to overpredict adverse emotional reactions to future events. People with high dysphoria symptoms (which include irritability, high stress, and negative emotions such as guilt, anger, hostility or melancholy, feeling defeated and overwhelmed, dissatisfaction, and lack of pleasure in everyday activities) report more frequent and more negatively valenced thoughts about their future, often perceived as unrealistic or implausible [68]. Research showed that an increased tendency to anticipate negative events in the future is related positively to anxiety and depression symptoms severity [66].

Self-efficacy and future anxiety were previously found as predictors of depression [32,36,69,71]. On the other hand, fostering self-efficacy in combat veterans had a positive impact on their outlook on the future and overall well-being [72,73,74]. However, little was known about these relationships among emerging adults [59]. This study filled this gap, showing that self-efficacy significantly contributes to depression and can explain its variability in 21%, while future anxiety adds another 15% to the understanding of this phenomenon. Therefore, training to improve self-efficacy and the affirmation of future achievements should be helpful in depression treatment [65,70].

The results of the current study can be useful for practitioners dealing with emerging adults so that they are aware of the common challenges people face in this stage of life. It could also guide mental health advocates (mental health prevention, health awareness campaigns) to focus on crucial issues young adults face, which increase their future anxi-ety. Two main practical recommendations that arise from this research are enhancing emerging adults’ self-efficacy by experiencing agency and teaching them to deal with neg-ative thoughts that otherwise could lead to spiraling into negative thought patterns using cognitive behavioral methods. Future anxiety levels should be routinely identified in young adults so that early interventions, such as mindfulness-based training and therapies, can be implemented to reduce anxiety, especially in young adults. Individuals with high levels of future anxiety should participate in therapies to prevent depression. Depending on the source of the future anxiety, trauma therapy (e.g., Eye Movement Desensitization and Reprocessing [EMDR]) or acceptance and commitment therapy [ACT] could be recommended for these individuals. In people with increased levels of depressive symptoms, pharmacological and psychological therapy based on Beck’s cognitive triad is also recommended. 

### 4.4. Limitation of the Study

The present research has certain limitations that impede the generalization of its outcomes. The reliance on self-reported questionnaires in this study may result in biased self-description. The participants may intentionally or unintentionally provide answers that portray them in a more favorable light. Another potential limitation of this study is that it utilized an online survey with a snowball sampling method, which may limit the generalization. Therefore, the results cannot be generalized to the whole adult population. Furthermore, this study did not differentiate between individuals in the emerging adulthood phase based on their educational or occupational status, family status, or other factors. In future research, it would be beneficial to consider the impact of economic circumstances and global events on the variables used in this study. Finally, the cross-sectional design of this study means that causal relationships should be treated with great caution. International and longitudinal studies are needed to verify the current findings.

## 5. Conclusions

The findings of this study highlight the critical nature of addressing depression and anxiety about the future among young adults. Especially emerging adults and women require psychological support to alleviate depression and future anxiety. Additionally, women should be the primary target group of interventions aimed at increasing self-efficacy. Furthermore, based on social cognitive theories, we showed the mechanism of depression in the adult Polish population. A low sense of self-efficacy increases fear of the future, which, in turn, increases symptoms of depression. This study contributes to existing knowledge and adds missing evidence on the mediating role of future anxiety on the association between self-efficacy and depression, confirming social cognitive theories. By implementing appropriate preventive measures and support systems, it is possible to alleviate the burden faced by emerging adults and women. Moreover, this investigation has made a significant contribution to the advancement of knowledge related to future anxiety in young adults, thereby expanding the scope of research in this area.

## Figures and Tables

**Table 1:** Characteristics of adults (*N* = 284).

Variable	Categories	Emerging (*n* = 157)		Middle-Aged (*n* = 126)	
		*n*	%	*n*	%
Age	*M*/*SD*	23.15	2.25	43.52	8.95
	Women	115	40.5	73	25.7
Gender	Men	42	14.8	53	18.7
	Other	1	0.4	0	0.0
	Village	23	8.1	21	7.4
	City up to 50,000 inhabitants	16	5.6	27	9.5
Place of residence	A city 50,000–250,000 inhabitants	42	14.8	38	13.4
	A city 250,000–500,000 inhabitants	33	11.6	30	10.6
	City above 500,000 inhabitants	44	15.5	10	3.5
	Primary	3	1.1	0	0.0
	Vocational	2	0.7	4	1.4
	Secondary	71	25.0	48	16.9
Education	Bachelor degree	56	19.7	20	7.0
	Master degree	26	9.2	54	19.0
	Single	63	22.2	25	8.8
Relationship status	In a relationship	95	33.5	101	35.6
	Student	40	14.1	1	0.4
Professional status	Student employed	78	27.5	7	2.5
	Employed	37	13.0	112	39.4
	Unemployed	3	1.1	6	2.1
Socioeconomic status	Insufficient	21	7.4	12	4.2
	Enough for basic needs	75	26.4	48	16.9
	Meets more than basic needs	62	21.8	66	23.2
Depression (PHQ-9)	Women (*M*/*SD*)	10.40	6.28	8.12	6.50
	Men (*M*/*SD*)	7.31	4.76	4.53	4.08
Future anxiety (DFS)	Women (*M*/*SD*)	19.40	7.77	16.22	8.56
	Men (*M*/*SD*)	14.64	7.60	12.34	8.85
Self-efficacy (GSES)	Women (*M*/*SD*)	27.16	6.13	28.86	5.50
	Men (*M*/*SD*)	32.50	4.84	33.00	4.63

**Table 2:** Descriptive statistics (*N* = 284).

Variable	Items	Range	*M*	*SD*	Skew.	Kurt.	ω	AVE	Pearson’s *r* Correlations		
									SE	FA	DS
Self-efficacy (SE)	10	11–40	29.48	6.02	–0.46	–0.20	0.92	0.64	0.73		
Future anxiety (FA)	5	0–30	16.56	8.55	–0.25	–1.00	0.91	0.71	–0.58 ***	0.82	
Depression symptoms (DS)	9	0–27	8.26	6.14	0.83	0.01	0.88	0.55	–0.55 ***	0.65 ***	0.68

Note. Skew. = skewness, Kurt. = kurtosis. The bold diagonal numbers of this table are the square root of the AVE. *** *p* < 0.001.

**Table 3:** Hierarchical regression analysis for depression symptoms (*N* = 284).

Step	Predictor	* b *	* SE *	95% CI		* t *	* R *	* R * ²	* F *	* df *_ 1 _	* df *_ 2 _
				LL	UL						
1	Intercept	4.71	0.67	3.40	6.03	7.06 ***	0.35	0.12	19.18 ***	2	281
	Age (Emerging)	2.48	0.70	1.10	3.85	3.55 ***					
	Gender (Women)	3.28	0.73	1.83	4.72	4.46 ***					
2	Intercept	21.67	1.89	17.94	25.40	11.44 ***	0.58	0.33	46.30 ***	3	280
	Age (Emerging)	1.79	0.62	0.58	3.00	2.92 **					
	Gender (Women)	0.94	0.69	–0.41	2.29	1.37					
	Self-efficacy	–0.51	0.05	–0.62	–0.40	–9.41 ***					
3	Intercept	8.81	2.22	4.44	13.18	3.96 ***	0.69	0.48	63.75 ***	4	279
	Age (Emerging)	1.15	0.55	0.07	2.23	2.10 *					
	Gender (Women)	0.74	0.61	–0.46	1.94	1.21					
	Self-efficacy	–0.25	0.06	–0.36	–0.14	–4.38 ***					
	Future anxiety	0.34	0.04	0.26	0.42	8.83 ***					

Note. CI = confidence interval, LL = lower level, UL = upper level. * *p* < 0.05, ** *p* < 0.01, *** *p* < 0.001.

**Table 4:** Parameter estimates for mediation model (*N* = 284).

				95% CI				
Predictor	Dependent	b	SE	LL	UL	β	z	*p*
SE	FA	–0.62	0.04	–0.70	–0.53	–0.67	–14.60	<0.001
FA	DS	0.56	0.06	0.45	0.68	0.62	9.50	<0.001
SE ⇒ FA ⇒ DS		–0.19	0.05	–0.29	–0.09	–0.23	–3.77	<0.001
Indirect effect		–0.35	0.04	–0.43	–0.26	–0.41	–8.05	<0.001

Note. FA = future anxiety, SE = self-efficacy, DS = depression symptoms, CI = confidence interval, LL = lower level, UL = upper level.

## Data Availability

The raw data supporting the conclusions of this article will be made available by the authors on request.
